# Disruption to diabetes and hypertension care during the COVID-19 pandemic in Latin America and the Caribbean and mitigation approaches: a scoping review

**DOI:** 10.1186/s12913-025-12760-3

**Published:** 2025-05-08

**Authors:** Oluwabunmi Ogungbe, Samira Barbara Jabakhanji, Roopa Mehta, John McCaffrey, David Byrne, Sinéad Hurley, Lori Rosman, Eyram Cyril Bansah, Folahan Ibukun, Irene Afua Quarshie, Katherine Lord, Yidan Lu, Yunzhi Wang, Asma Rayani, Hairong Liu, Ann Joseph, Alejandro Escobosa, Ivy Nyamuame, Jieun Lee, Ning Meng, Ibrahim Jehanzeb, Temitope Akinyemi, Shoichiro Nohara, Mauro F. F. Mediano, Yvette Yeboah-Kordieh, Cecilia de Sousa, Juliana Farhat, Renato Bandeira de Mello, Tara Taeed, Lawrence J. Appel, Sonia Y. Angell, Edward W. Gregg, Kunihiro Matsushita

**Affiliations:** 1 Johns Hopkins University School of Nursing, Baltimore, MD USA; 2 Department of Health, Behavior and Society, Johns Hopkins Bloomberg School of Public Health, Baltimore, MD USA; 3 School of Population Health, RCSI University of Medicine and Health Sciences, Dublin, Ireland; 4 Center for Preventive Medicine and Digital Health, Medical Faculty Mannheim, Heidelberg University, Heidelberg, Germany; 5 Departamento de Endocrinología y Metabolismo, Instituto Nacional de Ciencias Médicas y Nutrición, Salvador Zubirán, Mexico City, México; 6 Welch Medical Library, School of Medicine, Johns Hopkins University, Baltimore, MD USA; 7 Department of Epidemiology, Johns Hopkins Bloomberg School of Public Health, Baltimore, MD USA; 8 Welch Center for Prevention, Epidemiology and Clinical Research, Johns Hopkins University, 2024 E. Monument St., Suite 2-600, Baltimore, MD 21287 USA; 9 Johns Hopkins Carey Business School, Baltimore, MD USA; 10 Department of International Health, Johns Hopkins Bloomberg School of Public Health, Baltimore, MD USA; 11 Krieger School of Arts and Sciences, Johns Hopkins University, Baltimore, MD USA; 12 Department of Radiology, University of Medical Sciences, Havana, Cuba; 13 Department of Radiology, Ho Teaching Hospital, Volta Regional Hospital, Ho, Ghana; 14 Department of Internal Medicine, Rutgers/Community Medical Center, Toms River, NJ USA; 15 Evandro Chagas National Institute of Infectious Diseases, Oswaldo Cruz Foundation, Rio de Janeiro, RJ Brazil; 16 Department of Research and Education, National Institute of Cardiology, Rio de Janeiro, RJ Brazil; 17 Department of Internal Medicine, MedStar Health, Baltimore, MD USA; 18 Department of Internal Medicine, Federal University of Rio Grande Do Sul, Porto Alegre, Brazil; 19 Division of Cardiology, Johns Hopkins School of Medicine, Baltimore, USA

**Keywords:** Latin America, The Caribbean, Disruptions, COVID-19, Telemedicine, Chronic disease, Diabetes, Hypertension, Resilience, Pandemics

## Abstract

**Background:**

The COVID-19 pandemic disrupted care for non-communicable diseases globally. This study synthesizes evidence on disruptions to primary care, focusing on hypertension and diabetes care and mitigation approaches taken during the pandemic in Latin America and the Caribbean (LAC).

**Methods:**

We conducted a scoping review, searching nine electronic databases for studies from January 2020 to December 2022 on COVID-19-related primary care disruptions and interventions, including studies on hospital-based interventions given their relevance to the pandemic response in LAC. We adapted the Primary Health Care Performance Initiative framework to develop our search strategy and synthesize data. For studies reporting interventions, we included studies conducted outside of LAC.

**Results:**

Of 33,510 references screened, 388 studies were included (259 reported disruptions in LAC, 61 interventions in LAC, 63 interventions outside LAC, and five interventions from countries within and outside LAC), with three-quarters presenting data from Brazil, Argentina, Mexico, and Peru; few studies focused on rural areas. Additionally, the few studies that adequately quantified care disruptions reported a reduction in hypertension and diabetes control during the pandemic (e.g., hypertension control rate decreased from 68 to 55% in Mexico). Frequently reported causes of disruption included burnout and mental health challenges among healthcare workers (with disproportionate effects by type of worker), reduced medication supplies, and reduced frequency of clinic visits by patients (e.g., due to financial constraints). The most reported interventions included remote care strategies (e.g., smartphone applications, virtual meeting platforms) and mental health programs for healthcare workers. Remote care strategies were deemed feasible for care delivery, triaging, and clinical support for non-physicians. Patients were generally satisfied with telemedicine, whereas providers had mixed perceptions. Robust evidence on the effectiveness of remote care strategies for diabetes and hypertension care was unavailable in LAC.

**Conclusion:**

Hypertension and diabetes control appeared to worsen in LAC during the pandemic. Major reported causes of care disruptions were workforce issues, reduced medication supply, and changes in patient perceptions of seeking and receiving primary healthcare. Remote care strategies were feasible for various purposes and were well received by patients. However, the lack of data on intervention effectiveness underscores the importance of strengthening research capacity to generate robust evidence during future pandemics. Developing resilient healthcare systems able to provide care for hypertension and diabetes during future pandemics will depend on investment in the healthcare workforce, medical supply chain, health data and research infrastructure, and technology readiness.

**Supplementary Information:**

The online version contains supplementary material available at 10.1186/s12913-025-12760-3.

## Panel: Research in context

### What is already known on this topic

While evidence on health system impacts of the COVID19 pandemic and potential interventions has been emerging rapidly, comprehensive syntheses in Latin America and the Caribbean (LAC) were unavailable.

### What this study adds

Drawing from 388 studies on primary care disruptions in LAC and corresponding interventions in and outside of LAC, this scoping review describes pandemic disruptions and mitigation interventions for diabetes and hypertension care from 2020 to 2022. Differences were identified in the volume of studies describing care disruptions and interventions by country/geography, and by the domain of primary care provision. Most publications were from Brazil, Mexico, Argentina, Peru, and urban settings. Most disruptions were identified in the workforce, medication supplies, and patient perceptions of care. Mitigation responses predominantly included remote care strategies and mental health programs for the health workforce; however, robust data for their effectiveness in LAC were unavailable.

### How this study might affect research, practice or policy

This scoping review shows that action is needed to strengthen healthcare systems and their resilience in LAC. Research gaps remain in many countries and healthcare domains, a major gap being the lack of studies investigating intervention effectiveness. To provide care for persons with hypertension and diabetes during the next pandemic, countries in LAC should invest in the healthcare workforce, health data and research infrastructure, and technology readiness.

## Introduction

The COVID-19 pandemic disrupted care for non-communicable diseases (NCDs) like hypertension and diabetes (both type 1 and type 2 diabetes mellitus) around the world. A negative impact on the management of these conditions in Latin America and the Caribbean (LAC) was likely, as the region has some of the highest rates of chronic diseases globally, and a need for improvements to NCD care delivery was recognized before the pandemic [1–4]. Healthcare delivery in LAC operates through a mixed system of public facilities, social security institutions, and private providers [5]. Primary care is delivered through public health centers, social security clinics, and private practices, with varying levels of integration and coverage. In-service pharmacies, often located within healthcare facilities, play a crucial role in medication access. This fragmented system posed unique challenges during the pandemic response [6].

In 2021, Pan American Health Organization (PAHO) surveys across LAC found disruptions in the delivery of about half of essential healthcare services, including hypertension and diabetes management and prescriptions [7]. A study using household surveys in 14 LAC countries found that healthcare disruption was highest at the beginning of the pandemic in May–June 2020, with an average of 20.4% of households reporting disruption, and declined to 2.9% by May–July 2021 [8]. Furthermore, a qualitative study with health system decision-makers in eight LAC countries found that the main mitigation strategies focused on telemedicine, community-based care, expansion of hospital capacity and health workforce, and public–private collaborations. However, the magnitude of care disruptions and recovery approaches have yet to be systematically characterized [9].

We conducted a scoping review to answer two research questions: 1) To what extent were diabetes and hypertension care in the LAC region disrupted during the COVID-19 pandemic? and 2) Which interventions were shown to mitigate, reverse, or support the recovery of diabetes and hypertension care disruptions due to the pandemic? Our main focus was diabetes and hypertension since they are leading causes of mortality worldwide and often considered pathfinders for reforming primary care systems. Based on findings from the present scoping review, we provide potential actions to strengthen healthcare systems capable of providing care during future pandemics.

## Methods

This scoping review adheres to the PRISMA Extension for Scoping Reviews (Appendix 1 pp. 1–2) [10]. Before commencing this review, we modified the Primary Health Care Performance Initiative (PHCPI) Framework and used it to organize our analysis. The PHCPI Framework includes various domains (e.g., service delivery) and sub-domains (e.g., facility organization and management) of healthcare provision (Appendix 1 p. 3). We have published our study protocol [11] and summarized key review components below and in Table 1.

**Table 1:** Scoping review stages and description

Scoping Review Steps	Description
Conceptual framework	We adapted the Primary Health Care Performance Initiative Framework [11] [ (Appendix 1, p. 3)
Search strategy	Detailed in Reference 11, and search terms listed in Appendix 1 pp. 5-6
Inclusion/exclusion criteria	Detailed in Reference 11, and search terms listed in Appendix 1 p. 4
Title/abstract screening	Dual reviewers with a third reviewer if inconclusive screening
Full-text review	Dual reviewers with a third reviewer if inconclusive review
Data extraction	Dual reviewers with a third reviewer if inconclusive extraction (Appendix 1, pp. 7–8)
Quality assessment	Dual reviewers with a third reviewer if inconclusive quality assessment (Appendix 1 pp. 9–23)

### Search strategy and information sources

Guided by the Population, Concept, and Context framework and the modified PHCPI Framework (Appendix 1, p. 4), we iteratively developed the search strategy (Appendix 1, pp. 5–6) with an information specialist (L.R.). While the PHCPI framework focuses on primary care, we also included studies on hospital-based interventions, given their relevance to the pandemic response in LAC. We searched MEDLINE via PubMed, CINAHL, Global Health, Embase, Cochrane, Scopus, Web of Science, and LILACS (Latin American and Caribbean Health Sciences Literature) from January 1, 2020, until December 12, 2022. For grey literature, the World Bank and PAHO provided relevant policy documents and reports from their institutional repositories. Additionally, four regional content experts (selected by PAHO for their expertise in chronic disease management and health systems in LAC) reviewed our initial literature list and recommended additional grey literature sources from their respective countries (Barbados, Brazil, Mexico, and Peru [see Acknowledgments for the list]), focusing on policy documents, healthcare system reports, and institutional analyses not captured in scientific databases.

### Study selection and eligibility

After removing duplication [12, 13], two reviewers independently screened each title/abstract against specified criteria and reviewed potentially eligible full-text publications using Covidence® software. Conflicts were resolved by a third reviewer or team consensus. For the eligibility (Appendix 1, p. 4), the *population* of interest is all adults eligible for screening or receiving diabetes or hypertension care and children with diabetes or hypertension. The *concept* is a disruption to the delivery of primary care services and mitigation, reversal, or recovery of those services where interruptions occur. While our focus was on diabetes and hypertension care, we included broader primary care disruptions and interventions because these conditions are typically managed within comprehensive primary care systems. This allowed us to capture system-level changes that impacted chronic disease care delivery and to identify transferable intervention strategies, particularly given the limited disease-specific evidence available during the rapid pandemic response period. The *context* is LAC during the COVID-19 pandemic. For literature on interventions, we also included studies conducted outside LAC since their results may inform healthcare reform in LAC. Original studies, reports, and reviews with systematic search strategies in English, Spanish, or Portuguese were included.

### Data extraction

A data extraction and quality assessment template was developed and embedded in Covidence® to record study information and to map studies according to the modified PHCPI Framework (Appendix 1, pp. 7–8). Two independent reviewers extracted data and assessed study quality, and a third reviewer contributed to establishing consensus when needed. Studies were assessed for quality using the Mixed Methods Assessment Tool (MMAT) [14], due to the heterogeneity of study designs represented.

### Data synthesis

We synthesized study findings narratively. Given that telemedicine emerged as the predominant intervention type in our review (representing over 70% of interventions in LAC), we structured our analysis of intervention findings using established frameworks for evaluating telehealth implementations. This included assessing: (1) feasibility and utilization, (2) acceptability and satisfaction, and (3) effectiveness. Our analysis framework for interventions emerged from the data itself—telemedicine represented the predominant intervention type (LAC: *n* = 43, 71%; non-LAC countries: *n* = 52, 83%), warranting detailed categorization of these findings. For clarity, we present general disruption findings first, followed by a focused analysis of telemedicine interventions and narrative analysis of other types of interventions. MMAT results are displayed in a traffic plot using the *robvis* tool (Appendix 1, pp. 9–23) [15].

### Developing a list of potential actions

Together with PAHO, we established a panel of content experts from LAC. We shared preliminary study findings with the panel, World Bank, and PAHO representatives and received initial feedback in May/June 2023. Using the final scoping review findings, we developed a list of potential actions and refined these based on feedback from the expert panel, World Bank, and PAHO (See "Recommended actions" section). Thus, the recommended actions were systematically derived through a three-step process: 1) the authors drafted the recommendations based on the synthesis of evidence from the scoping review findings, particularly focusing on identified gaps and successful interventions to support interpretation based on their expertise of the regional context; 2) Regional experts, World Bank, and PAHO representatives provided initial feedback on preliminary literature findings; and 3) We refined the recommendations based on the experts’ input while ensuring each recommendation was supported by evidence from our review.

### Reflexivity

Our team includes authors who currently or have previously worked in LAC countries (RM, KL, AJ, AE, IN, TA, MFFM, CS, JF, RBM, SYA), as well as senior researchers with experience in international collaborations (KM, EWG, LJA, SYA). We approached this study with the intention of equitable inclusion of researchers with contextual knowledge of the region (Appendix 2) [16].

### Role of the funding source

This project was supported by the World Bank, and additional support to investigators was provided by Science Foundation Ireland. The expert panel was supported by PAHO. Although the World Bank, PAHO, and the expert panel had opportunities to review study materials and provide feedback, the content of the present study is solely the responsibility of the authors and may not represent the views of the World Bank, PAHO, Science Foundation Ireland, or the expert panel members.

## Results

**Fig. 1:**
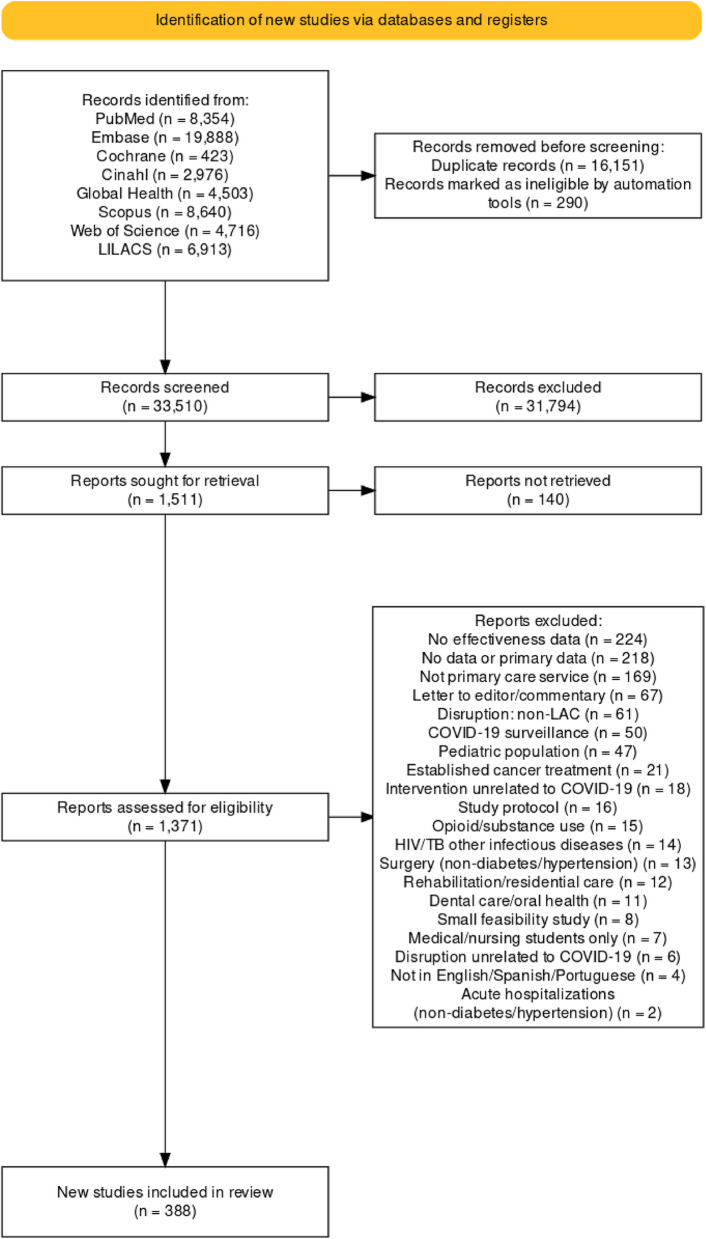
Selection of eligible studies, PRISMA flowchart. LAC =Latin America and the Caribbean; HIV/TB = Human Immunodeficiency Virus/Tuberculosis; LILACS = Latin American and Caribbean Health Sciences Literature

**Fig. 2:**
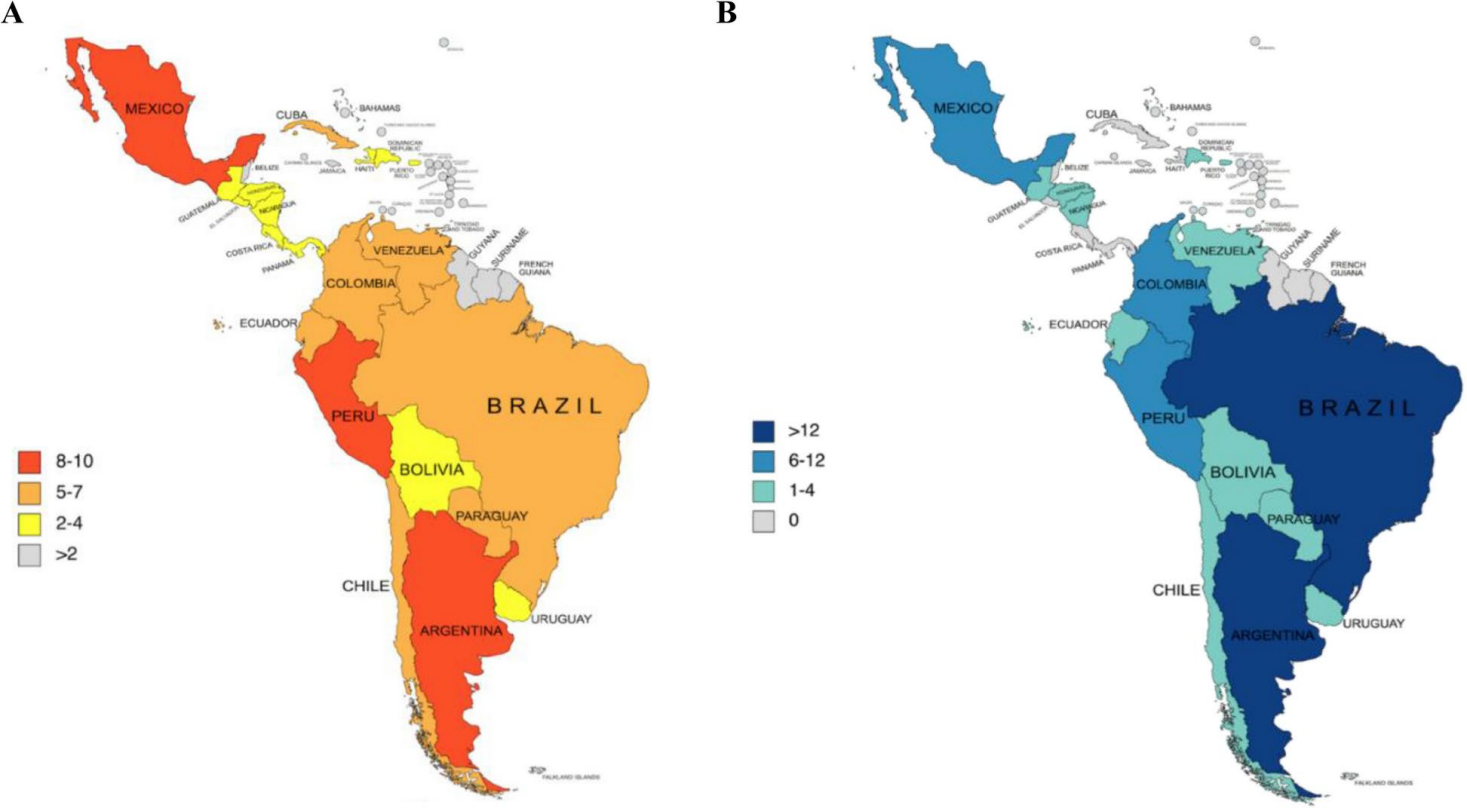
Geographical representation of included studies within Latin America and the Caribbean (LAC) on (**A**) disruptions to primary care; (**B**) interventions to mitigate disruptions

Our search identified 49,954 records (Fig. 1). After removing 16,441 duplicates, 33,510 titles/abstracts were screened for eligibility, and 1,511 remained for full-text review. Of these, 388 studies were included in this review, 259 reporting care disruptions in LAC and 129 exploring interventions within and outside LAC (Table 2, Appendix 1, p. 24). Of the 388 studies, most studies were from Brazil (*n* = 169), Argentina (*n* = 45), Mexico (*n* = 44), and Peru (*n* = 35); 59% (*n* = 228) were written in English only (Fig. 2, Table 2).

**Table 2:** Characteristics of included studies

	**Total**	^**c**^**Disruption**	^**d**^**Interventions (*N* = 129)**		
			**LAC**	**Non-LAC**	**Both**^**a**^
N	388	259	61	63	5
Publication Year, n (%)					
2020	41 (10.6)	31 (12.0)	8 (13.1)	1 (1.6)	1 (20.0)
2021	144 (37.1)	102 (39.4)	20 (32.8)	19 (30.2)	2 (40.0)
2022	203 (52.3)	126 (48.6)	33 (54.1)	43 (68.3)	2 (40.0)
Study Designs, n (%)					
Cross-sectional	189 (48.7)	155 (59.8)	18 (29.5)	14 (22.2)	2 (40.0)
Quasi/Pre-post	41 (10.6)	14 (5.4)	15 (24.6)	12 (19.0)	0 (0)
Cohort	37 (10.8)	20 (7.7)	8 (13.1)	8 (12.7)	1 (20.0)
Qualitative	40 (9.5)	28 (10.8)	6 (9.8)	6 (9.5)	0 (0)
RCT	7 (1.8)	0 (0)	3 (4.9)	4 (6.3)	0 (0)
Economic evaluation	1 (0.03)	0 (0)	0 (0)	1 (1.6)	0 (0)
Ecological/time-series analyses	14 (3.6)	13 (5.0)	0 (0)	1 (1.6)	0 (0)
Mixed methods	22 (5.4)	13 (5.0)	2 (3.3)	7 (11.1)	0 (0)
Review (scoping/systematic)	27 (5.7)	14 (5.4)	5 (8.2)	6 (9.5)	2 (40.0)
Other (audit, country profile, policy analyses)	10 (2.6)	2 (0.8)	4 (6.6)	4 (6.3)	0 (0.0)
Language, n (%)					
English	228 (58.8)	126 (48.6)	36 (59.0)	63 (100)	4 (80.0)
Spanish/Portuguese^b^	160 (41.2)	133 (51.4)	25 (41.0)	0 (0)	1 (20.0)

*LAC* Latin America and the Caribbean

^a^Both LAC and Non-LAC

^b^With or without English translations

^c^Disruption to the delivery of primary care services, with a particular focus on diabetes and hypertension care (awareness, detection, treatment and control), and recovery of those services where interruptions took place

^d^Interventions to recover potential disruptions in diabetes or hypertension care, including other primary care services in LAC (or other regions) during or after the pandemic. Numbers in parentheses represent percentages of total studies within each category

### Overview of studies on care disruption in LAC

Of 259 studies, 126 were from Brazil, 22 from Argentina, 22 from Mexico, and 19 from Peru (Appendix 1, p. 25). Where indicated, most studies described disruptions to primary care services in urban (*n* = 92) or mixed urban and rural settings (*n* = 69) and in public (*n* = 94) or mixed public and private healthcare settings (*n* = 69). Only five studies explicitly studied data in rural areas and only eight in private healthcare settings. Cross-sectional studies (*n* = 155) constituted the most common design, followed by qualitative (*n* = 28) and cohort studies (*n* = 20). Diabetes was explored in 41 studies (16%), and hypertension in 26 studies (10%) (Table 2, Appendix 1, p. 26).

**Fig. 3:**
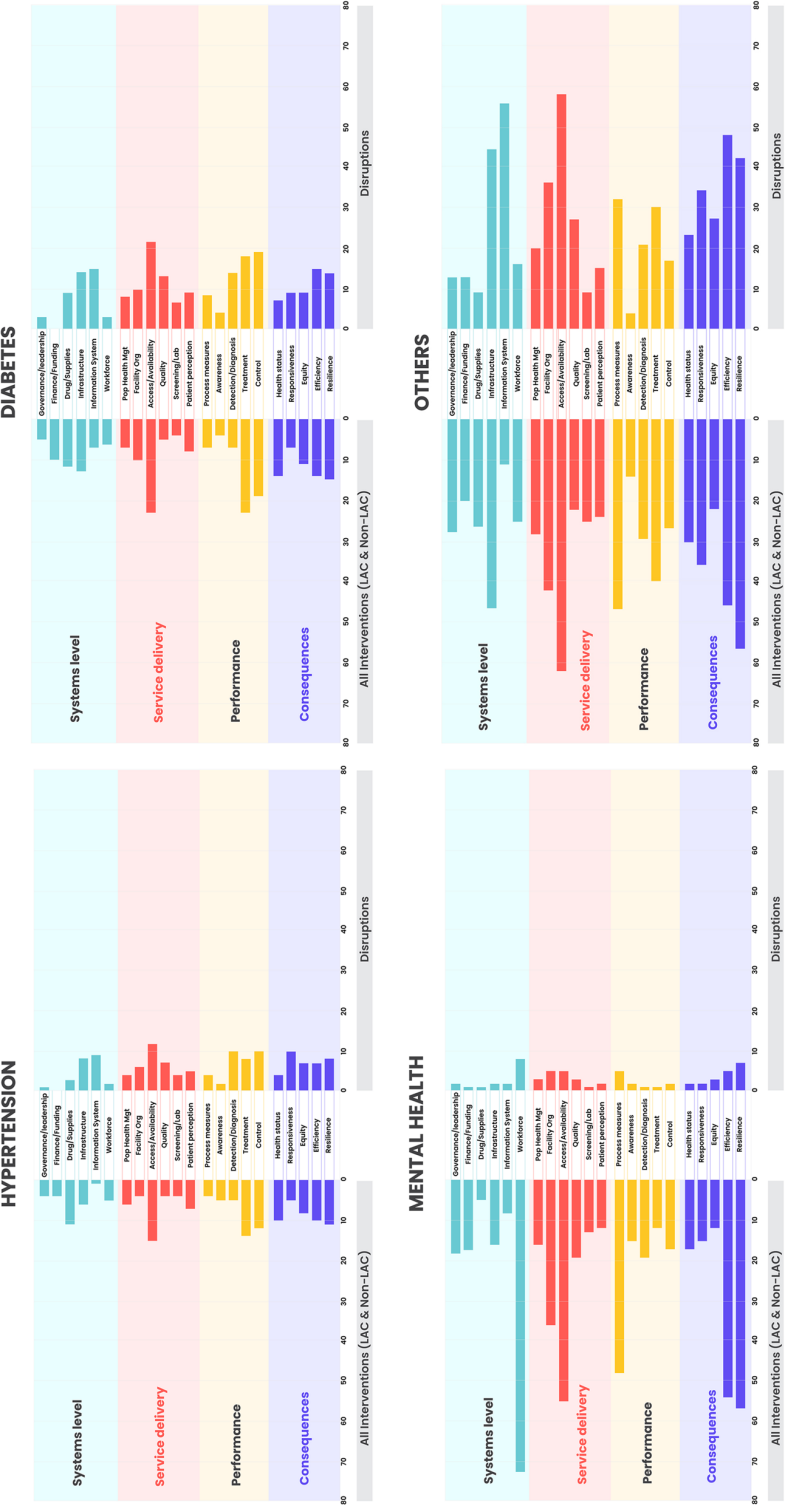
Domains of primary care services addressed in Latin America and the Caribbean (LAC) and Non-LAC countries, by disease area

Each sub-domain of the PHCPI Framework was represented in at least 20 papers (Fig. 3, Appendix 1, pp. 27–39). In the System or Inputs domains, workforce was studied most (*n* = 138), followed by facility infrastructure (*n* = 104), governance and leadership (*n* = 68), and drugs and supplies (*n* = 47). Within the Service Delivery domain, access to primary care services and availability of services were most frequently studied (*n* = 83), followed by patient perceptions of seeking and receiving primary healthcare and facility organization (*n* = 51). Within the Outputs domain, the most frequently studied elements were key process measures (*n* = 121, 31.2% of all studies), followed by hypertension or diabetes treatment and control metrics (*n* = 57, 14.7%). Studies most commonly addressed system resilience (*n* = 148, 38.1%) and responsiveness to people (*n* = 69, 17.8%) in the Outcomes domain. However, the coverage of domains and subdomains in Fig. 3 does not necessarily reflect the level of evidence for each due to variations in quality and objectivity of data (Figs. e2-e6, Tables e5 & e7).

#### Magnitude of disruptions to diabetes and hypertension care

Of 259 studies, diabetes was explored in 41 studies (16%), and hypertension in 26 studies (10%), only 12 studies (4.6%) provided quantitative data comparing treatment or control rates of diabetes and hypertension to pre-pandemic periods. The remaining studies examined broader health system disruptions affecting primary care delivery, including workforce capacity, medication supply chains, and healthcare access—factors directly impacting diabetes and hypertension management. Among these, a Chilean study examined national clinical data from sites implementing the World Health Organization HEARTS Technical Package [17] and reported a 12% and a 14% reduction in treatment and control rates of hypertension, respectively, in 2021 vs. 2017–2019. Similarly, social security data representing more than half of Mexico’s population [18] demonstrated a decrease in hypertension control rates from 68 to 55%. This same investigation showed a reduction in diabetes control rates, from 36 to 26% during the pandemic. Decreases in hypertension and diabetes screening, including its complications, were also reported [19, 20]. In Brazil, there was a decrease in guideline-recommended screening examinations among patients with type 1 diabetes from 23% pre-pandemic to under 4% in 2022 [21]. Decreasing clinic visits for diabetes and hypertension management were also reported in Chile, Haiti, and Mexico [22].

#### Major disrupted subdomains

##### Workforce

**Mental health of healthcare workers**

One hundred fifteen papers reported workforce-related mental health outcomes. Increases in burnout, anxiety, depression, and insomnia among doctors, nurses, and other healthcare staff were described in several countries [23–28]. Administrative data from Brazil showed a 17% increase in psychiatric sick leave during the pandemic [29]. Another Brazilian study noted a 39% increase in mental health absenteeism among 32,691 hospital workers [30]. Nursing assistants and other frontline workers were disproportionately impacted compared to physicians and nurses [31–33]. Contributing factors included lack of personal protective equipment (PPE), increased patient volume due to COVID-19, disruptions to workflow, social isolation, and perception of insufficient organizational and governmental support [23, 34–38]. A Colombian study reported that workers with an increased workload due to COVID-19 had ~ 2 times higher odds of anxiety and depression compared to those without workload changes [33]. Increased negative health behaviors, including smoking, alcohol use, and reduced physical activity, were also reported among healthcare workers [39].

**Changes in work environment**

During the pandemic, changes to the work environment and conditions were reported [40–43]. In Brazil, increases in physician income were noted at public facilities, whereas income was reduced in the private sector [41]. A Brazilian study showed increases in job availabilities for registered nurses (14%), nurse assistants (9%), physiotherapists (8%), and physicians (5%) [42]. A Brazilian survey of 1,609 healthcare workers found that 65% of psychologists were working remotely, while only 20% of dentists were doing so [43]. Moreover, while opportunities for early assumption of clinical responsibilities among young doctors in Brazil arose during the pandemic, challenges related to support and social integration of early career physicians were noted [44].

##### Medication and essential supplies

In a 2020 survey of 40 diabetes organizations (organizations responsible for advocacy and support of diabetes care in their respective countries) across 18 LAC countries, approximately two-thirds of respondents reported shortages of diabetes medications and a lack of critical supplies, such as syringes/needles and glucose strips. Only 37% of the respondents reported that a policy had been introduced to allow for the multi-month delivery of diabetes medications and supplies at home or dispensaries for two- to three months [45]. In Peru, availability of essential medicines like losartan and metformin declined initially, but rebounded in primary care, exceeding pre-pandemic levels. Recovery appeared lower, however, in secondary and tertiary care facilities [46]. Lack of PPE and other essential supplies were also reported in 15 studies (5.8% of disruption studies) [30, 40–42].

##### Access and availability of relevant services

Several studies highlighted substantial reductions in preventive and screening services, routine check-ups, and outpatient care in general [47–51]. For example, a Brazilian study reported a 77% drop in pap smears and a 43% decrease in mammograms in 2020–2021 compared to pre-pandemic levels. Significant declines in essential health services were reported in Mexico, including screenings (26–96% decrease), outpatient visits (9–40% decrease), and maternal health appointments (5–33% decrease) [18]. Outpatient services and elective procedures were also impacted, with decreases ranging from 46–75% across different studies [52–54]. Many facilities focused resources on pandemic response, resulting in barriers like long wait times for primary care [48, 55]. While several studies mentioned reductions in screening services, none specifically reported on screening for new cases of hypertension or diabetes.

##### Governance and leadership

The pandemic revealed gaps in governance and leadership across multiple levels of health systems in LAC [56–58]. Studies emphasized challenges in coordinating evidence-based responses, managing scarce resources, and confronting healthcare inequities exacerbated by the pandemic [59–63]. Significant regional disparities in health workforce availability were also reported [64]. Delays and inconsistencies in healthcare guidelines and policies were reported in Mexico during the initial pandemic stages [65]. One study reported that Brazilian municipalities perceived the municipal pandemic management as inadequate across various facets, including infrastructure, supplies, and communication [55].

##### Patient factors

Studies identified patient-level barriers to disease management, including worsened financial situations, fear of COVID-19 infection in clinics, and reduced adherence to medications and lifestyle changes [55, 66, 67]. A Mexican study found that over half of patients with self-reported health needs did not seek care because they considered their issue not severe; other reasons were costs and fear of COVID-19 infection [19]. Lifestyle changes also impacted disease management. A study in Brazil found that during the pandemic, eating habits worsened among adults with type 1 diabetes [68].

### Overview of intervention studies

Of 129 studies addressing interventions, 61 were carried out in LAC (Table 2), predominantly in Brazil (*n* = 28), Mexico (*n* = 11), Argentina (*n* = 10), and Colombia (*n* = 8) (Appendix 1, pp. 40–57, Fig. e11). Three studies included several LAC countries, and five included data from both LAC and non-LAC regions. Outside LAC, most interventions were from the United States (*n* = 38) and European countries (*n* = 9), and eight had multi-country representation.

In LAC, 30% of studies focused on diabetes (*n* = 18) and 16% on hypertension (*n* = 10) (Fig. 3). Other major investigated conditions included mental health (*n* = 12, 20%) and general medical encounters (*n* = 14, 23%). The corresponding numbers in non-LAC countries were as follows: diabetes (*n* = 13, 21%), hypertension (*n* = 6, 10%), mental health (*n* = 9, 14%), and general medical encounters (*n* = 23, 37%).

Two main types of interventions were implemented both in and outside LAC: remote care strategies, generally termed “telehealth” or “telemedicine” (LAC: *n* = 43, 71%; non-LAC countries: *n* = 52, 83%, Appendix 1, pp. 58–65), and mental healthcare (LAC: *n* = 12, 20%; non-LAC countries: *n* = 8, 13%, Appendix 1, pp. 66–67). Other interventions involved drug access and prescriptions, behavioral interventions, policy, diet, funding, screening campaigns, and team-based care. Interventions targeted either patients or healthcare workers.

### Remote care strategies within LAC

Of the 43 studies in LAC, half were cross-sectional and descriptive studies (*n*= 20); only three were randomized controlled trials (RCTs) [69–71]. Remote care was applied across numerous conditions, including 11 studies on diabetes and 5 on hypertension. Remote care was predominantly offered in tertiary care settings (*n* = 27; primary care: *n* = 7) delivered during real-time synchronous encounters (*n* = 35). Technologies used in the studies ranged from telephone calls (*n* = 20) to smartphone applications and virtual meeting platforms such as WhatsApp (*n* = 22), Zoom, and Microsoft Teams (*n* = 13). Studies evaluated various aspects of remote care implementation: feasibility and utilization (*n* = 31), healthcare provider or patient acceptance and satisfaction (*n* = 14), and some level of intervention effectiveness (*n* = 19).

#### Feasibility and utilization 

##### Healthcare delivery and clinical education

Studies reported feasibility based on the number of teleconsultations completed. Studies assessed feasibility through various metrics, primarily: number of completed teleconsultations (*n* = 18), technical success rates (*n* = 7), and ability to maintain scheduled appointments (*n* = 6). In Mexico, a primary-care diabetes clinic [72] and a large hospital-based study [73] perceived phone calls, text messaging, and video calls helpful for safe continuity of care (defined as maintaining regular patient monitoring and medication management through multidisciplinary telemedicine consultations, while avoiding disruption of treatment and preventing adverse events) and monitoring prescription needs among patients with diabetes or hypertension. In Brazil, telephone calls and text messages permitted monitoring of adherence among patients with type 1 diabetes [74]. Additionally, WhatsApp messages, audio, and video calls improved patients'access in remote communities [64]. A cross-sectional analysis of an ongoing randomized trial in Guatemala [75] reported on the success of telephone calls for verifying delivery of antihypertensive medication (e.g., 73% in the intervention group vs. 51% in the control group). Remote healthcare strategies were also used for patient education. In Colombia, web-based video conferencing was used to train patients with type 1 diabetes who were switching from regular insulin pumps to a hybrid closed-loop system [76]. In Brazil, telephone calls by nurses providing education on hypertension treatment and COVID-19 guidance helped avoid unnecessary emergency room visits [77].

##### Triaging and clinical support

A Brazilian study concluded that teleophthalmology for patients with diabetes was a feasible low-cost strategy to increase diabetic retinopathy screening in remote areas [78]. Similarly, in Peru, teleophthalmology was used to identify patients requiring face-to-face visits [79]. Other examples included WhatsApp video calls to triage for respiratory symptoms [80], telephone consultations to triage primary care patients to specialty care [81], and a remote medical advice program triaging patients requiring in-person hospital evaluation [73]. Another study from Mexico implemented teleconsultations to provide remote neurology support [82]. Over 95% of the 304 teleconsultations conducted by neurology residents successfully addressed neurological issues without needing an in-person evaluation.

#### Acceptance and satisfaction

##### Acceptance and satisfaction—patients

In Argentina [83], patients in a cardiology clinic rated virtual visits highly (e.g., easy to carry out and not inferior to face-to-face visits). Similar findings were reported among primary care and hospital-based patients in Chile [84]. In 56,560 web-based video consultations in Colombia [85], patients'satisfaction was excellent at 84%. Acceptance also was high (> ~ 85%) among neurology patients [82] and patients with diabetes [72]. Some studies reported that patients accepted virtual visits because of the pandemic, but generally preferred in-person visits [77, 86].

##### Acceptance and satisfaction– healthcare workers

A Brazilian study [87] found that while only 19% of providers had used telemedicine pre-pandemic, 83% reported engaging in telemedicine training, and 64% used telemedicine to provide care during the pandemic. However, perceptions of telehealth among healthcare professionals were mixed; in Chile, one study [84] reported that 62% of medical providers felt their clinical skills were challenged on video visits.

#### Effectiveness

Of 19 studies that aimed to evaluate effectiveness, most did not quantify disease outcomes or lacked control/comparator groups. Furthermore, some studies were small (e.g., < 50 participants). None conducted a health economic assessment.

##### Diabetes

A Colombian study using video conferences to monitor and train 91 patients with type 1 diabetes showed significant improvements in time-in-range glucose control, increasing from 77% to 82% when comparing pre- and post-intervention periods [76]. An observational study in Brazil carried out telephone monitoring of 237 patients with type 2 diabetes [74] and reported a small but statistically significant reduction in HbA1c (7.9% vs. 7.7%, *p* = 0.004). However, a study [88] (*n* = 143) that used telephone calls and text messages to send blood glucose values, check insulin doses, and periodically monitor for acute complications in patients with type 1 diabetes observed worsening of HbA1c in 46% of patients. A Brazilian center [89] (*n* = 747) assessed a telemedicine program without specific training and found hospitalization rates increased by ~ 50%. In contrast, a pre-post study from the Dominican Republic [90] used WhatsApp and telephone calls to monitor 946 dialysis patients and observed decreased hospitalization and a 2% COVID-19 diagnosis rate over a 3-month follow-up period.

##### Hypertension

A Chilean study used video calls from a mobile device to facilitate communication between the emergency department and a remote neurologist for rapid thrombolytic decision-making for stroke. Time from arrival to decision for thrombolytic administration was significantly reduced (median 5 [Interquartile Range, IQR: 3–8] vs. 6 [IQR 4–10] minutes, *p*= 0.016) compared to pre-implementation [91]. A study from Guatemala used an integrated intervention approach, including patient health coaching and home blood pressure monitoring in rural communities, and reported fewer problems with antihypertensive medication availability [75].

#### Team-based care strategies in LAC

Two RCTs within LAC utilized multi-professional team-based approaches for delivering remote interventions. An RCT (*n* = 91) in Brazil found that a 16-week multi-professional remote intervention, including weekly telephone calls and educational materials, significantly reduced the prevalence of positive screening for mental health disorders (37.0% vs. 57.8%, *p* = 0.04) and diabetes-related emotional distress (21.7% vs. 42.2%, *p*= 0.03) compared to standard care among adults with type 2 diabetes [71]. Another RCT (*n*= 47) evaluated the effects of a 16-week multi-professional remote intervention with a nurse compared to an in-person multi-professional intervention without a nurse on body composition parameters among adults with obesity in Brazil [69]. The remote intervention group had significant decreases in waist circumference (*p* < 0.001) and blood glucose (*p* = 0.014) compared to in-person care.

#### Remote care strategies outside the LAC region

We identified additional insights regarding intervention effectiveness in studies from countries outside LAC. For example, remote diabetes care delivered by primary care physicians in the USA achieved better medication adherence compared to non-physician providers (83% vs 77%) [92]. Additionally, HbA1c control rates were similar in urban settings (63% vs 65%), but remote care showed lower control rates in rural areas (56% vs 63%) [93–95]. Other studies indicated that rural areas face more barriers to engagement with virtual care options than urban areas [96–98]. In contrast, some studies found that telemedicine increased access and utilization for rural residents and other underserved populations in Canada [94], Saudi Arabia [82], and other countries [99]. Other barriers included insufficient technological literacy or skills and discomfort with remote monitoring devices [100]. Furthermore, internet availability, costs, reimbursement considerations, privacy or data concerns, and practice workflow challenges emerged as common themes [100]. One Australian study reported initial cost increases to the Medicare Benefits Schedule for general practitioner consultations after expansion of public telehealth coverage (pre-pandemic: $545 million per month; March 2020– December 2021: $592 million per month; *p*= 0.0001). After this initial increase, there was no significant change in costs [93].

### Mental health support for providers in LAC

In Uruguay [101], an online mindfulness program reported improved stress scores among 15 healthcare workers. A brief remote cognitive behavioral therapy intervention decreased anxiety and depression symptoms in 26 Mexican healthcare workers, though time constraints and workload impeded participation [102]. In Brazil [103], a program providing mental health support for 395 hospital-based healthcare workers showed that workers appreciated the support and connectedness from the virtual interventions. An RCT (*n*= 120) in Brazil showed that oral cannabidiol (CBD) plus standard care vs standard care alone reduced emotional exhaustion over a month. CBD also reduced anxiety and depression symptoms, but five serious adverse events occurred in the CBD group [70].

### Mental health support for providers outside LAC

An RCT in Turkey showed reduced stress and work-related strain and improved psychological well-being among 52 nurses after mindfulness-based breathing and music therapy [104]. Similarly, a mindfulness-based course lowered workforce distress, emotional exhaustion, fear of COVID-19, and physical stress symptoms, including sleep difficulties in Italy [105]. In France, a psychological support hotline was rapidly implemented to provide support for hospital workers during the COVID-19 outbreak. After 26 days, the hotline had received 149 calls, helped assess symptoms, and referred staff to additional psychosocial support [106].

### Other interventions

#### Interventions within LAC

A digital COVID19 screening protocol among over 500 healthcare workers in Mexico promoted workforce wellness and NCD prevention and control by identifying previously undiagnosed hypertension, diabetes, and obesity [54, 107]. A 10-month medication home delivery program through partnerships with local pharmacies for over 800 older adults in Brazil resulted in 25% fewer in-person visits to the pharmacy. Among the program users, 67% and 21% received medications for hypertension and diabetes, respectively, and 92% of users considered the program satisfactory [108].

#### Interventions outside LAC

In the USA, an analysis of electronic health records for 2,479 cardiometabolic patients showed that pharmacist-led medication review interventions (e.g., simplifying complex regimes) helped conserve supplies, reduce staff exposure, and maintain patient safety [109]. In Portugal, a cross-sectional study of 603 patients found that community pharmacies taking on dispensing of typically hospital-only medications improved therapy adherence and lowered patient travel and absenteeism [110].

In Saudi Arabia, a prospective interventional study delivered pharmacy-based education to 54 patients with diabetes, which improved disease knowledge, self-care, medication adherence, and diabetes control compared to usual care [111]. In the USA, a community-based participatory approach providing online lifestyle education to 105 participants in underserved communities significantly improved health behaviors, self-reported health, and mental health scores [112]. Integration of diabetes screening into routine COVID-19 testing facilities for 923 low-income individuals in a cross-sectional study identified high rates of previously undiagnosed diabetes and connected patients to follow-up care [113]. Similarly, an RCT using non-clinical patient coaches increased cancer screening rates among 119 patients compared to usual care.

### Recommended actions

Based on the findings from this scoping review and feedback from the World Bank, PAHO, and our expert panel, we list potential actions relevant to LAC in Table 3. While the relevance of these findings will vary across LAC countries, they aim to contribute to strengthening data infrastructure for real-time evaluation of care disruptions and improve the resilience and equity of healthcare systems.

**Table 3:** Recommended actions based on review findings

Domain	Potential Actions
System (Sub-domains: Governance & Leadership, Healthcare Financing, Population Health Needs, Surveillance, Innovation & Learning	• Invest in telehealth infrastructure and equitable, virtual health outreach • Create “learning communities” to share best practices between countries, for example in relation to telehealth adoption and chronic disease management • Invest in nationwide healthcare data systems and qualified personnel, to enable real-time analysis allowing for earlier detection of disruptions to non-communicable disease care • Establish standardized measures and reporting for monitoring health indicators, including service utilization and health outcomes, and metric critical to detecting health inequities, to enable benchmarking, comparisons across geographies and time, economic analysis, and relevant and responsive systems changes • Strengthen cross-regional coordination and collaboration on health system resilience planning through agencies like the Pan-American Health Organization
Input (Sub-domains: Drugs & Supplies, Facility Infrastructure, Information Systems, Workforce, Funds	• Implement mental health promotion initiatives, screening programs, counseling and forums to support workforce resiliency • Institutionalize evidence-based self-care skills training to aid coping mechanisms among healthcare workers • Integrate team-based care strategies into care models (e.g. involving pharmacists, community health workers, lay health workers) • Create a roster of retired healthcare workers for emergency situation • Develop policy specifying expanded roles of healthcare workers in emergency situations, including those working in the private healthcare sector • Diversify import sources and increase local production of drugs and medical supplies to avoid shortages during crises • Enable cross-national collaborations on sourcing, stockpiling and distribution of essential medicines, e.g. antihypertensives and diabetes medications
Service Delivery (Sub-domain: Population Health Management, Facility Organization & Management, Access, Availability of Effective Healthcare Services	• Scale remote primary care strategies like telemedicine and related essential infrastructure to maintain continuity when in-person care is disrupted • Expand reach of essential services into hard-to-access and rural communities through telehealth, including digital literacy programs and technical support services • Leverage user-friendly, inclusive digital tools like telehealth consultations and mHealth applications to increase community awareness and equitable access to care • Implement patient-centered treatment models tailored to community priorities and resources to improve engagement in self-care behaviors • Harness digital health tools to increase access to medications and lifestyle change support needed to optimize treatment success

The domains of Output and Outcomes are not included in this table since they are downstream of System, Input, and Service Delivery and responses are likely to aim at these three upstream domains

## Discussion

Our scoping review of 388 articles found some evidence that hypertension and diabetes care services in LAC were disrupted during the COVID-19 pandemic and indicated potential pathways that may explain these disruptions. Workforce (including the mental health of healthcare workers) and medication supplies were the most frequently reported disrupted domains in LAC. Disruptions to the healthcare environment were exacerbated at the patient level, resulting in adverse lifestyle changes, financial constraints, and fear of contracting COVID-19.

Our review also identified several promising mitigating interventions. Remote care strategies, including telemedicine (e.g., telephone calls, text messaging, video conferences), teleophthalmology, medication home delivery programs, and mental health care of healthcare workers were the two most studied interventions in LAC. Remote care strategies facilitated healthcare service delivery, clinical support of healthcare workers, and patient education, and they appeared feasible and acceptable. However, knowledge gaps remain in long-term benefits and cost-effectiveness of remote care in LAC. Findings reveal a breadth of innovations from several countries to mitigate pandemic disruptions, but rigorous evaluation is needed, particularly on sustainability and integration within health systems.

Given the number of studies identified in our scoping review, it was surprising that only a few evaluated changes in hypertension or diabetes control during the pandemic. This was likely a product of limited capacity for immediate assessment of relevant data in care delivery settings and the redirection of staff and system’s focus on the COVID-19 response. Introducing data infrastructure and sufficient workforce capacity to allow for NCD treatment and control surveillance and data analysis in real-time could address these gaps.

Our review revealed substantial pandemic impacts on health workforces across LAC (e.g., burnout and mental distress), with disproportionate effects on some types of workers, e.g., worse for nurse assistants compared to physicians [36]. It is natural for healthcare systems to focus on patient needs, but functional healthcare systems require a healthy workforce. Relevant also in non-LAC countries [32], this scoping review signaled several areas LAC countries could consider for strategies to improve the well-being of healthcare workers, such as monitoring provider distress, workload redistribution with task sharing policies, and increasing access to mental health resources [36]. Dedicated efforts will also be required to build robust emergency response capacities, such as developing rosters of retired workers to be mobilized for emergencies and policies enabling extended task-shifting across provider cadres in emergencies [114].

While medication shortages occurred initially in some settings, supplies rebounded quickly in others, contrasting with more prolonged global supply chain disruptions [115]. These differences may be related to domestic pharmaceutical production capacity and supply chain resilience. Countries like Brazil reported pronounced insulin shortages [116], while Peru's rebound aligned with policies fortifying local manufacturing after past shortages [46]. Diversifying import sources, localized production and patient-centered delivery models can help countries prepare supply chains to withstand future crises. Cross-national regional collaborations on sourcing, stockpiling, and distribution may also be highly effective.

The pandemic introduced several unique circumstances leading to changes in patient behavior potentially detrimental to hypertension and diabetes management. For example, fear of COVID-19 infection posed a major barrier to care-seeking. Patients also faced intensified financial constraints and occupational disruptions (e.g., unemployment), that may have impacted care-seeking and self-management [117]. Studies also revealed worsening mental health, diet, and health behaviors during lockdowns—all likely exacerbating existing illness. Remote care addressed some of these patient-level factors and indeed emerged as an adaptive strategy, as detailed below.

Most studies in LAC suggested that remote care strategies helped maintain continuity and access across a spectrum of clinical care needs. More specifically, telehealth enabled remote monitoring for clinical decision-making and helped reduce potential COVID-19 exposure. Patient acceptance was generally high, whereas provider acceptance was lower, highlighting the need for more telemedicine training in medical education. Although a few studies reported that telehealth performs comparably to in-person primary care [72, 89, 118], the evidence remains limited regarding its clinical effectiveness, cost-effectiveness, and long-term sustainability [119, 120]. Also, structural barriers around health literacy and digital skills (particularly among elderly patients), insufficient availability of technology, limited infrastructure, and suboptimal connectivity (particularly in rural and poorer regions) can be issues. There may also be cost-related barriers (e.g., out-of-pocket costs) [67, 75, 121]. These findings highlight the value of patient-centered telehealth in addressing these structural barriers [98]. While global collaborations can accelerate learnings on how to optimize remote care strategies, optimal design (e.g., telehealth modalities like video conferences) of remote care and evaluation frameworks tailored to LAC contexts will be essential for scaling up remote care models in the region [122].

### Proposed modification to the conceptual framework

**Fig. 4:**
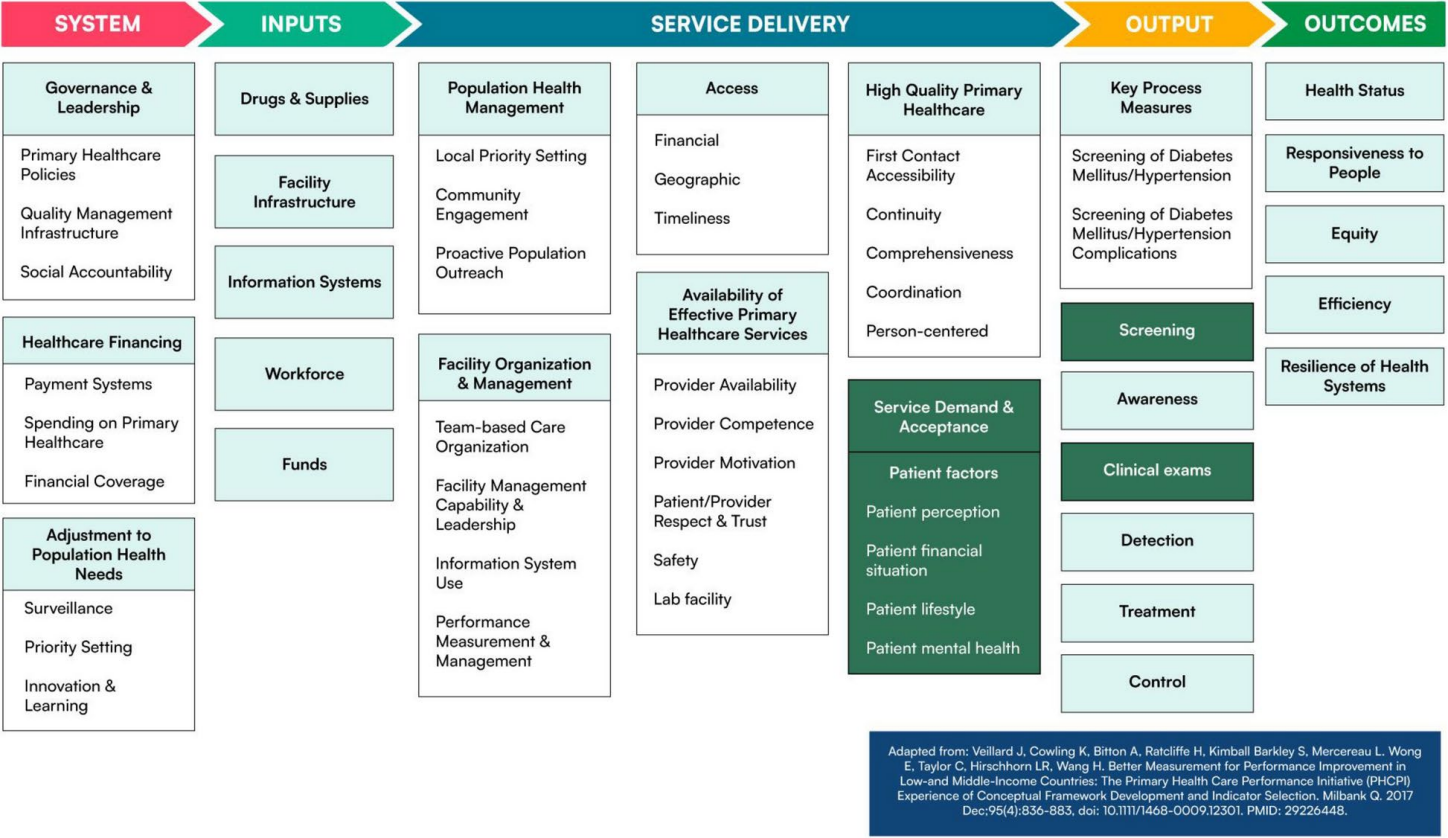
Second modification of the Primary Health Care Performance Initiative Conceptual Framework based on scoping review findings. Dark green boxes indicate framework amendments

After completing this scoping review, we perceived screening and lab testing in the ‘service delivery’ domain as closely linked to the domain of ‘outputs’ along with other diagnostic tests (e.g., imaging). Regarding patient factors, our initial conceptual framework only specified patient perception, whereas other patient-related elements (e.g., patient financial situation) are relevant. Thus, we propose modifying our conceptual framework for future studies, as depicted in Fig. 4.

### Limitations

Despite its comprehensiveness and rigor, the present scoping review has limitations. First, the limited data on interventions likely reflect the challenges of conducting rigorous research during the COVID-19 pandemic, especially in settings with limited research and data infrastructure, as well as the timing of our study (i.e., < 3 years into the pandemic). Second, as noted above, grey literature was not systematically searched. Third, we did not consider the timing of included studies with the spread of COVID-19 across specific LAC countries. Furthermore, the methodological quality of included studies varied considerably, and studies quantifying disruptions often lacked pre-pandemic comparison data or control groups. Our review yielded substantial heterogeneity across and within domains of interventions and contexts, making detailed subgroup analyses infeasible beyond broad comparisons across geography, conditions (diabetes vs hypertension), and healthcare delivery domains. Additionally, while our review included evidence from non-LAC regions, direct comparisons were limited by fundamental differences in health system structures, particularly the more robust pre-pandemic data infrastructure and monitoring systems available in many high-income countries compared to LAC regions. Finally, the review was restricted to studies published in English, Spanish, or Portuguese, while a few LAC countries have other languages as their primary language.

## Conclusions

This review revealed concerning disruptions to essential NCD services, including diabetes and hypertension care across LAC. Workforce capacity and well-being, medication supplies, and patient-level barriers (including fear of COVID-19 infection, financial constraints, and changed health-seeking behaviors) were the most frequently reported factors disrupting care delivery. While this review identified promising strategies for adapting service delivery, predominantly remote care strategies, gaps remain in their cost-effectiveness and sustainability in the region. Remote care interventions showed feasibility and acceptability, particularly in urban settings, though implementation challenges persisted in rural areas. Our findings highlight the critical need for strengthening healthcare systems through investment in workforce support, data infrastructure, and technological readiness. Future pandemic preparedness in LAC will require addressing these foundational elements while ensuring equitable access to care across all populations. These insights may inform health leaders in building resilient, equitable NCD care systems capable of maintaining essential services during future health emergencies.

## Supplementary Information

Supplementary Material 1.

Supplementary Material 2.

## Data Availability

Data is provided within the manuscript or supplementary information files.

## References

[1] Hartmann-Boyce J, Highton P, Rees K, et al. The impact of the COVID-19 pandemic and associated disruptions in health-care provision on clinical outcomes in people with diabetes: a systematic review. Lancet Diabetes Endocrinol. 2024;12(2):132–48. 10.1016/S2213-8587(23)00351-0. 38272607 10.1016/S2213-8587(23)00351-0

[2] Hennis AJM, Coates A, Del Pino S, et al. COVID-19 and inequities in the Americas: lessons learned and implications for essential health services. Rev Panam Salud Publica Pan Am J Public Health. 2021;45:e130. 10.26633/RPSP.2021.130. 10.26633/RPSP.2021.130 PMC8713468 34987555

[3] Hambleton IR, Caixeta R, Jeyaseelan SM, Luciani S, Hennis AJM. The rising burden of non-communicable diseases in the Americas and the impact of population aging: a secondary analysis of available data. Lancet Reg Health– Am. 2023;21. 10.1016/j.lana.2023.100483. 10.1016/j.lana.2023.100483 PMC10090658 37065858

[4] Msemburi W, Karlinsky A, Knutson V, Aleshin-Guendel S, Chatterji S, Wakefield J. The WHO estimates of excess mortality associated with the COVID-19 pandemic. Nature. 2023;613(7942):130–7. 10.1038/s41586-022-05522-2. 36517599 10.1038/s41586-022-05522-2 PMC9812776

[5] Ruano AL, Rodríguez D, Rossi PG, Maceira D. Understanding inequities in health and health systems in Latin America and the Caribbean: a thematic series. Int J Equity Health. 2021;20(1):94. s12939–021–01426–1. 10.1186/s12939-021-01426-1. 33823879 10.1186/s12939-021-01426-1 PMC8023548

[6] Cristian A Herrera, Jeremy Veillard, Nicole Feune de Colombi, Sven Neelsen, Geoff Anderson, Katherine Ward. Building Resilient Health Systems in Latin American and the Caribbean: Lessons Learned from the COVID-19 Pandemic. World Bank; 2022. https://documents1.worldbank.org/curated/en/099805001182361842/pdf/P1782990d657460cb0a2080ac0048f8b98f.pdf.

[7] World Health Organization (WHO). Second Round of the National Pulse Survey on Continuity of Essential Health Services during the COVID-19 Pandemic. January-March 2021.; 2021. https://www.who.int/publications/i/item/WHO-2019-nCoV-EHS-continuity-survey-2021.1. Accessed 6 Dec 2023.

[8] Herrera CA, Kerr AC, Dayton JM, Kakietek JJ. Healthcare service disruption in 14 Latin American and Caribbean countries during the COVID-19 pandemic: Analysis of household phone surveys, 2020–2021. J Glob Health. 2023;13:06023. 10.7189/jogh.13.06023. 37469284 10.7189/jogh.13.06023 PMC10359759

[9] Herrera CA, Juárez-Ramírez C, Reyes-Morales H, et al. COVID-19 Disruption To Routine Health Care Services: How 8 Latin American And Caribbean Countries Responded: The study examines disruption to routine health care services brought about by the COVID-19 pandemic in eight Latin American and Caribbean countries. Health Aff (Millwood). 2023;42(12):1667–74. 10.1377/hlthaff.2023.00694. 38048493 10.1377/hlthaff.2023.00694

[10] The Primary Health Care Performance Initiative. The PHCPI conceptual framework. https://improvingphc.org/phcpi-conceptual-framework. Accessed 26 Dec 2023.

[11] Jabakhanji SB, Ogungbe O, Angell SY, et al. Disruption of diabetes and hypertension care during the COVID-19 pandemic and recovery approaches in the Latin America and Caribbean region: a scoping review protocol. BMJ Open. 2024;14(1):e074443. 10.1136/bmjopen-2023-074443. 38262656 10.1136/bmjopen-2023-074443 PMC10806801

[12] Covidence systematic review software. www.covidence.org.

[13] The EndNote Team. EndNote. Published online 2013.

[14] Hong QN, Fàbregues S, Bartlett G, Boardman F, Cargo M, Dagenais P, Gagnon M-P, Griffiths F, Nicolau B, O’Cathain A, Rousseau M-C, Vedel I, Pluye P. The Mixed Methods Appraisal Tool (MMAT) version 2018 for information professionals and researchers. Education for Information. 2018;34(4):285-91.

[15] McGuinness LA, Higgins JPT. Risk-of-bias VISualization (robvis): An R package and Shiny web app for visualizing risk-of-bias assessments. Res Synth Methods. 2021;12(1):55–61. 10.1002/jrsm.1411. 32336025 10.1002/jrsm.1411

[16] Morton B, Vercueil A, Masekela R, et al. Consensus statement on measures to promote equitable authorship in the publication of research from international partnerships. Anaesthesia. 2022;77(3):264–76. 10.1111/anae.15597. 34647323 10.1111/anae.15597 PMC9293237

[17] Barake F, Paccot M, Rivera M, Neira C, Reyes V, Escobar MC. Chile’s public healthcare sector hypertension control rates before and during the pandemic and HEARTS implementation. Rev Panam Salud Publica. 2022;46:e126. 10.26633/rpsp.2022.126. 36071920 10.26633/RPSP.2022.126 PMC9440732

[18] Doubova SV, Leslie HH, Kruk ME, Pérez-Cuevas R, Arsenault C. Disruption in essential health services in Mexico during COVID-19: an interrupted time series analysis of health information system data. BMJ Glob Health. 2021;6(9). 10.1136/bmjgh-2021-006204. 10.1136/bmjgh-2021-006204 PMC8413469 34470746

[19] Colchero MA, Gómez R, Pineda-Antúnez CJ, Bautista-Arredondo SA. Health care utilization during the Covid-19 pandemic in Mexico: the cascade of care. Salud Publica Mex. 2021;63(6, Nov-Dic):743–750. 10.21149/12894. 10.21149/12894 35099906

[20] Carro GV, Carlucci EM, Torterola I, Breppe P, Ticona Ortiz MÁ, Palomino Pallarez JE. Diabetic foot and COVID-19. Medical consultation and severity of lesions compared to 2019. Med BAires. 2020;80(supl.6):30–4. 33481730

[21] Foppa L, Alessi J, Nemetz B, de Matos R, Telo GH, Schaan BD. Quality of care in patients with type 1 diabetes during the COVID-19 pandemic: a cohort study from Southern Brazil. Diabetol Metab Syndr. 2022;14(1):75. 10.1186/s13098-022-00845-6. 35598019 10.1186/s13098-022-00845-6 PMC9123820

[22] Arsenault C, Gage A, Kim MK, et al. COVID-19 and resilience of healthcare systems in ten countries. Nat Med. 2022;28(6):1314–24. 10.1038/s41591-022-01750-1. 35288697 10.1038/s41591-022-01750-1 PMC9205770

[23] Silvera Carminati AE, Prol Misura SM, Gallardo Denis YV. Situación de carga física y mental en enfermería de Uruguay durante la pandemia Covid 19. Rev Urug Enferm. 2022;17(2):1–20. 10.33517/rue2022v17n2a4.

[24] Bonilla-Sierra P, Manrique GA, Hidalgo-Andrade P, Ruisoto P. Psychological Inflexibility and Loneliness Mediate the Impact of Stress on Anxiety and Depression Symptoms in Healthcare Students and Early-Career Professionals During COVID-19. Front Psychol. 2021;12:729171. 10.3389/fpsyg.2021.729171. 34621223 10.3389/fpsyg.2021.729171 PMC8491304

[25] Cortés-Álvarez NY, Vuelvas-Olmos CR. COVID 19: Psychological Effects and Associated Factors in Mexican Nurses. Disaster Med Public Health Prep. 2022;16(4):1377–83. 10.1017/dmp.2020.495. 33706845 10.1017/dmp.2020.495 PMC7985626

[26] Gir E, Baptista CJ, Reis RK, Menegueti MG, Pillon SC, de Oliveira ESAC. Increased use of psychoactive substances among Brazilian health care professionals during the COVID-19 pandemic. Arch Psychiatr Nurs. 2022;41:359–67. 10.1016/j.apnu.2022.09.004. 36428073 10.1016/j.apnu.2022.09.004

[27] Mota IA, Oliveira Sobrinho GD, Morais IPS, Dantas TF. Impact of COVID-19 on eating habits, physical activity and sleep in Brazilian healthcare professionals. Arq Neuropsiquiatr. 2021;79(5):429–36. 10.1590/0004-282x-anp-2020-0482. 34037163 10.1590/0004-282X-ANP-2020-0482 PMC9394556

[28] Scatularo CE, Battioni L, Bellia S, et al. Psychophysical impact of the covid-19 pandemic on healthcare workers in argentina. The imppacts-sac.20 survey. Rev Argent Cardiol. 2021;89(3):196–202. 10.7775/rac.v89.i3.20231.

[29] Marconi AM, Myers U, Retamar AM, Freddi IJ, Chiarelli J, Zamora R. Increase in use of psychiatric sick leave during COVID-19 pandemic by healthcare workers in a municipality in Argentina. Rev Bras Med Trab. 2022;20(1):36–44. 10.47626/1679-4435-2022-872. 36118070 10.47626/1679-4435-2022-872 PMC9444219

[30] Barros-Areal AF, Albuquerque CP, Silva NM, et al. Impact of COVID-19 on the mental health of public university hospital workers in Brazil: A cohort-based analysis of 32,691 workers. PLoS ONE. 2022;17(6):e0269318. 10.1371/journal.pone.0269318. 35709187 10.1371/journal.pone.0269318 PMC9202958

[31] Dos Santos MA, Pereira FH, De Souza CJ, Oliveira HC, Ceolim MF, Andrechuk CRS. Sleep and Professional Burnout in Nurses, Nursing Technicians, and Nursing Assistants During the COVID-19 Pandemic. J Nurs Res Lippincott Williams Wilkins. 2022;30(4):e218–e218. 10.1097/jnr.0000000000000501. 10.1097/jnr.0000000000000501 PMC9301687 35674665

[32] Lotta G, Fernandez M, Correa M. The vulnerabilities of the Brazilian health workforce during health emergencies: analysing personal feelings, access to resources and work dynamics during the COVID-19 pandemic. (Special Issue: Global health and health workforce development: education, ma. Int J Health Plann Manage. 2021;36(s1):42–57. 10.1002/hpm.3117. 33502795 10.1002/hpm.3117 PMC8013198

[33] Llop-Gironés A, Vračar A, Llop-Gironés G, et al. Employment and working conditions of nurses: where and how health inequalities have increased during the COVID-19 pandemic? Hum Resour Health. 2021;19(1):112. 10.1186/s12960-021-00651-7. 34530844 10.1186/s12960-021-00651-7 PMC8444178

[34] Peñaranda A, García E, Pérez-Herrera LC, et al. Effect of the COVID-19 pandemic on the mental health, daily and occupational activities among health professionals in Colombia: a national study. BMC Psychiatry. 2022;22(1):682. 10.1186/s12888-022-04337-9. 36333782 10.1186/s12888-022-04337-9 PMC9635125

[35] Osorio-Martínez ML, Malca-Casavilca M, Condor-Rojas Y, Becerra-Bravo MA, Ruiz RE. Factors associated with the development of stress, anxiety and depression in the context of COVID-19 pandemic in Peruvian healthcare facilities. Arch Prev Riesgos Labor. 2022;25(3):271–84. 10.12961/aprl.2022.25.03.04. 36265109 10.12961/aprl.2022.25.03.04

[36] Franco JA, Leví P. Feelings, Stress, and Adaptation Strategies of Nurses against COVID-19 in Guayaquil. Invest Educ Enferm. 2020;38(3). 10.17533/udea.iee.v38n3e07. 10.17533/udea.iee.v38n3e07 PMC7885538 33306897

[37] Juarez-Garcia A, Camacho-Avila A, Garcia-Rivas J, Gutierrez-Ramos O. Psychosocial factors and mental health in Mexican healthcare workers during the COVID-19 pandemic. Salud Ment. 2021;44(5):229–40. 10.17711/sm.0185-3325.2021.030.

[38] Zipf AL, Polifroni EC, Beck CT. The experience of the nurse during the COVID-19 pandemic: A global meta-synthesis in the year of the nurse. J Nurs Sch. 2022;54(1):92–103. 10.1111/jnu.12706. 10.1111/jnu.12706 PMC8662101 34738314

[39] Bazán PR, Azevedo Neto RM, Dias JA, et al. COVID-19 information exposure in digital media and implications for employees in the health care sector: findings from an online survey. Einstein Sao Paulo. 2020;18:eAO6127. 10.31744/einstein_journal/2020AO6127. 10.31744/einstein_journal/2020AO6127 PMC7690931 33295429

[40] Cotrin P, Moura W, Gambardela-Tkacz CM, et al. Healthcare Workers in Brazil during the COVID-19 Pandemic: A Cross-Sectional Online Survey. Inquiry. 2020;57:46958020963711. 10.1177/0046958020963711. 33034257 10.1177/0046958020963711 PMC7550936

[41] de Oliveira B, Andrietta LS, Reis RS, et al. The Impact of the COVID-19 Pandemic on Physicians’ Working Hours and Earnings in São Paulo and Maranhão States, Brazil. Int J Env Res Public Health. 2022;19(16). 10.3390/ijerph191610085. 10.3390/ijerph191610085 PMC9408582 36011716

[42] Bigoni A, Malik AM, Tasca R, et al. Brazil’s health system functionality amidst of the COVID-19 pandemic: An analysis of resilience. Lancet Reg Health Am. 2022;10:100222. 10.1016/j.lana.2022.100222. 35284904 10.1016/j.lana.2022.100222 PMC8896985

[43] Campos J, Martins BG, Campos LA, de Fátima V-D, Marôco J. Symptoms related to mental disorder in healthcare workers during the COVID-19 pandemic in Brazil. Int Arch Occup Env Health. 2021;94(5):1023–32. 10.1007/s00420-021-01656-4. 33559748 10.1007/s00420-021-01656-4 PMC7871020

[44] Montagna E, Donohoe J, Zaia V, et al. Transition to clinical practice during the COVID-19 pandemic: A qualitative study of young doctors’ experiences in Brazil and Ireland. BMJ Open. 2021;11(9). 10.1136/bmjopen-2021-053423. 10.1136/bmjopen-2021-053423 PMC8460523 34551956

[45] Barone MTU, Villarroel D, de Luca PV, et al. COVID-19 impact on people with diabetes in South and Central America (SACA region). Diabetes Res Clin Pr. 2020;166:108301. 10.1016/j.diabres.2020.108301. 10.1016/j.diabres.2020.108301 PMC7332429 32623036

[46] Tenorio-Mucha J, Lazo-Porras M, Zafra J, Ewen M, Beran D. Using government data to understand the use and availability of medicines for hypertension and diabetes: lessons from Peru. J Pharm Policy Pr. 2022;15(1):86. 10.1186/s40545-022-00481-5. 10.1186/s40545-022-00481-5 PMC9675072 36401297

[47] de Degani GL, Duarte L, Ismael J, Martinez L, López F. The impact of the COVID-19 pandemic on cancer care in the public health subsector, province of Santa Fe. Argentina Ecancermedicalscience. 2021;15:1270. 10.3332/ecancer.2021.1270. 34567255 10.3332/ecancer.2021.1270 PMC8426009

[48] Duarte MBO, Argenton JLP, Carvalheira JBC. Impact of COVID-19 in Cervical and Breast Cancer Screening and Systemic Treatment in São Paulo, Brazil: An Interrupted Time Series Analysis. JCO Glob Oncol. 2022;8:e2100371. 10.1200/go.21.00371. 35696624 10.1200/GO.21.00371 PMC9225667

[49] Lopez-Huamanrayme E, Salsavilca-Macavilca E, Taype-Rondan A. Impact of the COVID-19 pandemic on outpatient endocrinology consultation and teleconsultation in a Peruvian hospital. Rev Cuerpo Medico Hosp Nac Almanzor Aguinaga Asenjo. 2022;15(3). 10.35434/rcmhnaaa.2022.153.1407.

[50] Rojas-Zumaran V, Walttuoni-Picón E, Campos-Siccha G, Cruz-Gonzales G, Huiza-Espinoza L, Moya-Salazar J. Decline of cytology-based cervical cancer screening for COVID-19: a single-center Peruvian experience. Medwave. 2022;22(10):e2589. 10.5867/medwave.2022.S3.2589. 36427327 10.5867/medwave.2022.S3.2589

[51] Tachibana BMT, Ribeiro RLM, Federicci É EF, et al. The delay of breast cancer diagnosis during the COVID-19 pandemic in São Paulo, Brazil. Einstein Sao Paulo. 2021;19:eAO6721. 10.31744/einstein_journal/2021AO6721. 10.31744/einstein_journal/2021AO6721 PMC8687645 34932776

[52] Horta BL, Silveira MF, Barros AJD, et al. COVID-19 and outpatient care: a nationwide household survey. Cad Saude Publica. 2022;38(4):e00194121. 10.1590/0102-311x00194121. 35442261 10.1590/0102-311X00194121

[53] Bozovich GE, Alves De Lima A, Fosco M, et al. Collateral damage of COVID-19 pandemic in private healthcare centres of Argentina. Med B Aires. 2020;80:37–41. 32658846

[54] Dimperio H, Gagliardi J, Zoni R, et al. Resultados de la Encuesta COVID-19 Impacto en la atención cardiovascular del Registro Nacional de Infarto ARGEN IAM-ST. Rev Argent Cardiol. 2020;88(3):222–30. 10.7775/rac.es.v88.i3.18150.

[55] Duarte LS, Shirassu MM, Atobe JH, de Moraes MA, Bernal RTI. Continuidade da atenção às doenças crônicas no estado de São Paulo durante a pandemia de Covid-19. Saúde Debate. 2021;45(spe2):68–81. 10.1590/0103-11042021e205.

[56] Mamani-Benito O, Esteban RFC, Ventura-León J, Caycho-Rodríguez T, Solís RF, Shocosh DHB. Effect of concern about COVID-19 on professional self-efficacy, psychological distress, anxiety, and depression in Peruvian health personnel. Salud Ment. 2021;44(5):215–20. 10.17711/SM.0185-3325.2021.028.

[57] Medina Guillen LF, Quintanilla Ferrufino GJ, Juárez Pérez I, Asfura JS. Occupational exposure to covid-19 in healthcare workers from Latin America, May 2020. Rev Cientif Cienc Med. 2020;23(2):207–13.

[58] Antiporta DA, Bruni A. Emerging mental health challenges, strategies, and opportunities in the context of the COVID-19 pandemic: Perspectives from South American decision-makers. Rev Panam Salud Publica. 2020;44:e154. 10.26633/rpsp.2020.154. 33245299 10.26633/RPSP.2020.154 PMC7679046

[59] Villalobos Dintrans P, Maddaleno M, Granizo Román Y, et al. [Disruption of health services for pregnant women, newborns, children, adolescents, and women during the COVID-19 pandemic: ISLAC 2020 ProjectInterrupção dos serviços de saúde para grávidas, recém-nascidos, crianças, adolescentes e mulheres durante a pand. Rev Panam Salud Publica. 2021;45:e140. 10.26633/rpsp.2021.140. 34737772 10.26633/RPSP.2021.140 PMC8559667

[60] Zafra-Tanaka JH, Najarro L, Tenorio-Mucha J, et al. COVID-19’s impact on type 1 diabetes management: A mixed-methods study exploring the Peruvian experience. Int J Health Plann Manage. 10.1002/hpm.3536. Published online 2022. 10.1002/hpm.3536 PMC9349690 35790022

[61] Villain P, Carvalho AL, Lucas E, et al. Cross-sectional survey of the impact of the COVID-19 pandemic on cancer screening programs in selected low- and middle-income countries: Study from the IARC COVID-19 impact study group. Int J Cancer. 2021;149(1):97–107. 10.1002/ijc.33500. 33533501 10.1002/ijc.33500 PMC8014228

[62] Steinman M, de Sousa JHB, Tustumi F, Wolosker N. The burden of the pandemic on the non-SARS-CoV-2 emergencies: A multicenter study. Am J Emerg Med. 2021;42:9–14. 10.1016/j.ajem.2020.12.080. 33429189 10.1016/j.ajem.2020.12.080 PMC7775794

[63] Luciani S, Agurto I, Caixeta R, Hennis A. Prioritizing noncommunicable diseases in the Americas region in the era of COVID-19. Rev Panam Salud PublicaPan Am J Public Health. 2022;46. 10.26633/RPSP.2022.83. 10.26633/RPSP.2022.83 PMC9299393 35875322

[64] dos Santos AL, Manzano M, Krein A. Heterogeneity in the distribution of health professionals in Brazil and the COVID-19 pandemic. (Desenvolvimento, saude e mudanca estrutural: o complexo economico-industrial da saude 4.0 no contexto da Covid-19.) [Portuguese]. Cad Desenvolv. 2021;16(28):197–219.

[65] Bautista-González E, Werner-Sunderland J, Pérez-Duarte Mendiola P, et al. Health-care guidelines and policies during the COVID-19 pandemic in Mexico: A case of health-inequalities. Health Policy Open. 2021;2:100025. 10.1016/j.hpopen.2020.100025. 33521627 10.1016/j.hpopen.2020.100025 PMC7836807

[66] de Pádua BR, Avila GO, Ritter AC, et al. Healthcare of pregnant women with diabetes during the COVID-19 pandemic: a Southern Brazilian cross-sectional panel data. J Perinat Med Published online. 2022. 10.1515/jpm-2022-0177. 10.1515/jpm-2022-0177 36398907

[67] Braghieri HA, Correia M de A, Carvalho JF de, et al. Impacto da pandemia da COVID-19 sobre o tratamento medicamentoso dos pacientes com doença arterial periférica: um estudo observacional transversal. J Vasc Bras. 2021;20:e20210021-e20210021. 10.1590/1677-5449.210021.

[68] de Oliveira GB, Alessi J, Erthal IN, et al. Healthy lifestyle gone bad: effect of the COVID-19 pandemic on the daily habits of children and adolescents with type 1 diabetes. Arch Endocrinol Metab Online. 2022;66(3):345–54. 10.20945/2359-3997000000490. 10.20945/2359-3997000000490 PMC9832844 35657126

[69] Christinelli HCB, Westphal G, Costa MAR, Okawa RTP, Nardo Junior N, Fernandes CAM. Multiprofessional intervention and telenursing in the treatment of obese people in the COVID-19 pandemic: a pragmatic clinical trial. Rev Bras Enferm. 2022;75(supl.2):e20210059-e20210059. 10.1590/0034-7167-2021-0059. 10.1590/0034-7167-2021-0059 35476094

[70] Crippa JAS, Zuardi AW, Guimarães FS, et al. Efficacy and Safety of Cannabidiol Plus Standard Care vs Standard Care Alone for the Treatment of Emotional Exhaustion and Burnout Among Frontline Health Care Workers During the COVID-19 Pandemic: A Randomized Clinical Trial. JAMA Netw Open. 2021;4(8):e2120603. 10.1001/jamanetworkopen.2021.20603. 34387679 10.1001/jamanetworkopen.2021.20603 PMC8363917

[71] Alessi J, de Oliveira GB, Franco DW, et al. Telehealth strategy to mitigate the negative psychological impact of the COVID-19 pandemic on type 2 diabetes: A randomized controlled trial. Acta Diabetol. 2021;58(7):899–909. 10.1007/s00592-021-01690-1. 33723649 10.1007/s00592-021-01690-1 PMC7959296

[72] Silva-Tinoco R, Torre-Saldaña V de la. La imperiosa necesidad de telemedicina en la atención de diabetes durante la pandemia de COVID-19. Un estudio de abordaje integral. Gac Méd Méx. 2021;157(3):323–326. 10.24875/gmm.20000674. 10.24875/GMM.M21000563 34667324

[73] Mendoz RL, Moreno GYC, Martinez Arredondo HA, et al. Remote Healthcare Program in Mexico in the Context of the COVID-19 Pandemic. Heal Inf Res. 2022;28(2):152–9. 10.4258/hir.2022.28.2.152. 10.4258/hir.2022.28.2.152 PMC9117807 35576983

[74] de Mattos Matheus AS, Cabizuca CA, Tannus LRM, et al. Telemonitoring type 1 diabetes patients during the COVID-19 pandemic in Brazil: was it useful? Arch Endocrinol Metab. 2021;65(1):105–11. 10.20945/2359-3997000000309. 33166438 10.20945/2359-3997000000309 PMC10528697

[75] Hernández-Galdamez D, Mansilla K, Peralta AL, et al. Monitoring Study Participants and Implementation with Phone Calls to Support Hypertension Control During the COVID-19 Pandemic: The Case of a Multicomponent Intervention Trial in Guatemala. Glob Heart. 2021;16(1):77. 10.5334/gh.954. 34900568 10.5334/gh.954 PMC8622336

[76] Gómez AM, Henao D, Parra D, et al. Virtual training on the hybrid close loop system in people with type 1 diabetes (T1D) during the COVID-19 pandemic. Diabetes Metab Syndr. 2021;15(1):243–7. 10.1016/j.dsx.2020.12.041. 33450533 10.1016/j.dsx.2020.12.041 PMC7785279

[77] Correia DM da S, Pimentel ACE, Costa LBD da, et al. Teleorientação a hipertensos resistentes durante a pandemia por COVID-19: uma ação inovadora na enfermagem. Enferm Foco Brasília. 2020;11(2,n.esp):179–184. 10.21675/2357-707X.2020.v11.n2.ESP.3860.

[78] Queiroz MS, de Carvalho JX, Bortoto SF, et al. Diabetic retinopathy screening in urban primary care setting with a handheld smartphone-based retinal camera. Acta Diabetol. 2020;57(12):1493–9. 10.1007/s00592-020-01585-7. 32748176 10.1007/s00592-020-01585-7 PMC7398859

[79] Huancahuari Ayala G, Roman-Benate JF, Hernandez Peña A, Ticse AR. Teleorientation in the ophthalmology service in a public hospital from May to August 2020 during the COVID-19 pandemic in Peru. Acta Medica Peru. 2022;39(2):120–7. 10.35663/amp.2022.392.2322.

[80] Carneiro AC, de Pinho GS, Belo JV, et al. Outcomes of telemedicine care during the COVID-19 pandemic: Experience from an intervention program designed for vulnerable population in Brazil. J Telemed Telecare. 10.1177/1357633X221089151. Published online 2022:1357633X221089151. 10.1177/1357633X221089151 PMC8948534 35321612

[81] Sperling S, Andretta CRL, Basso J, et al. Telehealth for Supporting Referrals to Specialized Care During COVID-19. Telemed J E Health. 2022;28(4):544–50. 10.1089/tmj.2021.0208. 34314637 10.1089/tmj.2021.0208

[82] Domínguez-Moreno R, García-Grimshaw M, Chávez-Martínez OA, et al. Global & Community Health: Implementation of and Patient Satisfaction With the First Neurologic Telemedicine Program in Mexico During COVID-19. Neurology. 2021;97(6):293–6. 10.1212/wnl.0000000000012291. 34045275 10.1212/WNL.0000000000012291

[83] Burgos LM, Benzadón M, Candiello A, et al. Telehealth in Heart Failure Care during COVID-19 Pandemic Lockdown in Argentina. Int J Heart Fail. 2020;2(4):247–53. 10.36628/ijhf.2020.0025. 36262173 10.36628/ijhf.2020.0025 PMC9536725

[84] Garcia-Huidobro D, Rivera S, Valderrama Chang S, Bravo P, Capurro D. System-Wide Accelerated Implementation of Telemedicine in Response to COVID-19: Mixed Methods Evaluation. J Med Internet Res. 2020;22(10):e22146. 10.2196/22146. 32903195 10.2196/22146 PMC7541041

[85] Escobar MF, Henao JF, Prieto D, et al. Teleconsultation for outpatient care of patients during the Covid-19 pandemic at a University Hospital in Colombia. Int J Med Inf. 2021;155:104589. 10.1016/j.ijmedinf.2021.104589. 10.1016/j.ijmedinf.2021.104589 PMC8453779 34592540

[86] Loza CA, Baez G, Valverdi R, et al. A qualitative study on the elderly and accessibility to health services during the COVID-19 lockdown in Buenos Aires, Argentina - Part 2. Medwave. 2021;21(4):e8192. 10.5867/medwave.2021.04.8192. 34086668 10.5867/medwave.2021.04.8192

[87] Aquino E, Domingues RB, Mantese CE, Fantini F, Nitrini R, Prado GFD. Telemedicine use among neurologists before and during COVID-19 pandemic. Arq Neuropsiquiatr. 2021;79(7):658–64. 10.1590/0004-282x-anp-2020-0488. 34231649 10.1590/0004-282X-ANP-2020-0488

[88] Santana YE, Liberatore RDRJ. Teleconsultation for pediatric patients with type 1 diabetes mellitus during the COVID-19 pandemic: experience of a university hospital in Brazil. J Pediatr Rio J. 2022;98(6):587–9. 10.1016/j.jped.2022.02.001. 35276099 10.1016/j.jped.2022.02.001 PMC8885301

[89] Tabuti NIM, Pellizzari C, Carrascossi H, et al. Impact of telemedicine on metabolic control and hospitalization of peritoneal dialysis patients during the COVID-19 pandemic: a national multicentric cohort study. J Bras Nefrol Published online. 2022. 10.1590/2175-8239-jbn-2021-0113. 10.1590/2175-8239-JBN-2021-0113 PMC9838680 35199824

[90] Polanco E, Aquey M, Collado J, et al. A COVID-19 pandemic-specific, structured care process for peritoneal dialysis patients facilitated by telemedicine: Therapy continuity, prevention, and complications management. Ther Apher Dial. 2021;25(6):970–8. 10.1111/1744-9987.13635. 33634948 10.1111/1744-9987.13635 PMC8014150

[91] Delfino C, Mazzon E, Cavada G, et al. A Chilean Experience of Telestroke in a COVID-19 Pandemic Year. Cerebrovasc Dis. 2022;51(5):690–4. 10.1159/000523920. 35390787 10.1159/000523920 PMC9148905

[92] Baughman DJ, Jabbarpour Y, Westfall JM, et al. Comparison of Quality Performance Measures for Patients Receiving In-Person vs Telemedicine Primary Care in a Large Integrated Health System. JAMA Netw Open. 2022;5(9):e2233267. 10.1001/jamanetworkopen.2022.33267. 36156147 10.1001/jamanetworkopen.2022.33267 PMC9513647

[93] De Guzman KR, Snoswell CL, Caffery LJ, Wallis KA, Smith AC. Costs to the Medicare Benefits Schedule for general practitioner consultations: A time-series analysis. J Telemed Telecare. 2022;28(10):726–32. 10.1177/1357633x221122135. 36346935 10.1177/1357633X221122135

[94] Ryan BL, Brown JB, Freeman TR, et al. Virtual family physician care during COVID-19: a mixed methods study using health administrative data and qualitative interviews. BMC Prim Care. 2022;23(1):300. 10.1186/s12875-022-01902-9. 36434524 10.1186/s12875-022-01902-9 PMC9700898

[95] Lee LC, Farwig P, Kirk L, Mitchell VD, Sabatino JA, Barnes KD. Impact of pharmacist intervention on anticoagulation management and risk for potential COVID-19 exposure during the COVID-19 pandemic. Thromb Res. 2022;217:52–6. 10.1016/j.thromres.2022.07.004. 35868151 10.1016/j.thromres.2022.07.004 PMC9288238

[96] Vieira Silva CRD, Lopes RH, de Goes Bay O Jr, et al. Digital Health Opportunities to Improve Primary Health Care in the Context of COVID-19: Scoping Review. JMIR Hum Factors. 2022;9(2). 10.2196/35380. 10.2196/35380 PMC9159467 35319466

[97] Adepoju OE, Chae M, Liaw W, Angelocci T, Millard P, Matuk-Villazon O. Transition to telemedicine and its impact on missed appointments in community-based clinics. Ann Med. 2022;54(1):98–107. 10.1080/07853890.2021.2019826. 34969330 10.1080/07853890.2021.2019826 PMC8725902

[98] Chang JE, Lai AY, Gupta A, Nguyen AM, Berry CA, Shelley DR. Rapid Transition to Telehealth and the Digital Divide: Implications for Primary Care Access and Equity in a Post-COVID Era. Milbank Q. 2021;99(2):340–68. 10.1111/1468-0009.12509. 34075622 10.1111/1468-0009.12509 PMC8209855

[99] Scheffer M, Cassenote A, de Britto EAM, Russo G. The multiple uses of telemedicine during the pandemic: the evidence from a cross-sectional survey of medical doctors in Brazil. Glob Health. 2022;18(1):81. 10.1186/s12992-022-00875-9. 10.1186/s12992-022-00875-9 PMC9483882 36123696

[100] Kruse C, Heinemann K. Facilitators and Barriers to the Adoption of Telemedicine During the First Year of COVID-19: Systematic Review. J Med Internet Res. 2022;24(1). 10.2196/31752. 10.2196/31752 PMC8729874 34854815

[101] Castellano GS, Tarasenko YN. An on-line mindfulness-based stress reduction program for health workers in Uruguay amidst the COVID-19 pandemic April 2020. Psychiatry Res. 2022;313. 10.1016/j.psychres.2022.114599. 10.1016/j.psychres.2022.114599 PMC9071193 35526423

[102] Robles R, Ascencio L, Díaz D, et al. Implementation Science of Telepsychotherapy for Anxiety, Depression, and Somatization in Health Care Workers Dealing with COVID-19. Telemed J E Health Published online. 2022. 10.1089/tmj.2022.0155. 10.1089/tmj.2022.0155 36126309

[103] Fukuti P, Uchôa CLM, Mazzoco MF, et al. COMVC-19: A Program to protect healthcare workers’ mental health during the COVID-19 Pandemic. What we have learned. Clin Sao Paulo. 2021;76:e2631. 10.6061/clinics/2021/e2631. 10.6061/clinics/2021/e2631 PMC8579850 34817044

[104] Yıldırım D, Çiriş YC. The Effect of Mindfulness-Based Breathing and Music Therapy Practice on Nurses’ Stress, Work-Related Strain, and Psychological Well-being During the COVID-19 Pandemic: A Randomized Controlled Trial. Holist Nurs Pract. 2022;36(3):156–65. 10.1097/HNP.0000000000000511. 35435877 10.1097/HNP.0000000000000511 PMC8997019

[105] Marotta M, Gorini F, Parlanti A, Berti S, Vassalle C. Effect of Mindfulness-Based Stress Reduction on the Well-Being, Burnout and Stress of Italian Healthcare Professionals during the COVID-19 Pandemic. J Clin Med. 2022;11(11). 10.3390/jcm11113136. 10.3390/jcm11113136 PMC9180958 35683520

[106] Geoffroy PA, Le Goanvic V, Sabbagh O, et al. Psychological Support System for Hospital Workers During the Covid-19 Outbreak: Rapid Design and Implementation of the Covid-Psy Hotline. Front Psychiatry. 2020;11. 10.3389/fpsyt.2020.00511. 10.3389/fpsyt.2020.00511 PMC7326137 32670100

[107] Ortiz Contreras J, Quiroz Carreño JM, Neira Contreras R, et al. Sistematización de iniciativas en salud sexual y reproductiva según criterios de buenas prácticas en repuesta a la pandemia COVID-19 en la atención primaria en Chile. Medwave. 2022;22(6):e002555–e002555. 10.5867/medwave.2022.06.002555. 35917236 10.5867/medwave.2022.06.002555

[108] Alves JS, Gonçalves AMS, Bittencourt MN, Alves VM, Mendes DT, Nóbrega M. Psychopathological symptoms and work status of Southeastern Brazilian nursing in the context of COVID-19. Rev Lat Am Enferm. 2022;30:e3518. 10.1590/1518-8345.5768.3518. 10.1590/1518-8345.5768.3518 PMC9015705 35319627

[109] Burgess LH, Cooper MK, Wiggins EH, et al. Utilizing Pharmacists to Optimize Medication Management Strategies During the COVID-19 Pandemic. J Pharm Pract. 2022;35(2):184–9. 10.1177/0897190020961655. 33016180 10.1177/0897190020961655 PMC9086205

[110] Murteira R, Romano S, Teixeira I, Galante H, Sousa S, Teixeira RA. POSA247 Impact of Transferring the Dispensing of Hospital-Only Medicines to Community Pharmacies during COVID-19 Pandemic: A Single-Arm, Before-and-After Study. Value Health. 2022;25(1):S157. 10.1016/j.jval.2021.11.762. 10.1016/j.jval.2022.03.004 PMC9002297 35428552

[111] Khan YH, Alzarea AI, Alotaibi NH, et al. Evaluation of Impact of a Pharmacist-Led Educational Campaign on Disease Knowledge, Practices and Medication Adherence for Type-2 Diabetic Patients: A Prospective Pre- and Post-Analysis. Int J Env Res Public Health. 2022;19(16):12. 10.3390/ijerph191610060. 10.3390/ijerph191610060 PMC9408490 36011692

[112] Iglesias A, Ambrose A, Coronel-Mockler S, et al. Impact of transitioning to virtual delivery of a cardiovascular health improvement program for Latinos during the COVID-19 pandemic. BMC Public Health. 2022;22(1):1935. 10.1186/s12889-022-14291-6. 36258185 10.1186/s12889-022-14291-6 PMC9579581

[113] Kerkhoff AD, Rojas S, Black D, et al. Integrating Rapid Diabetes Screening Into a Latinx Focused Community-Based Low-Barrier COVID-19 Testing Program. JAMA Netw Open. 2022;5(5):e2214163. 10.1001/jamanetworkopen.2022.14163. 35616939 10.1001/jamanetworkopen.2022.14163 PMC9136625

[114] World Health Organization. Working for Health 2022–2030 Action Plan. 2022. https://creativecommons.org/licenses/by-nc-sa/3.0/igo/. Accessed 29 Jan 2024.

[115] Bown CP, Bollyky TJ. How COVID-19 vaccine supply chains emerged in the midst of a pandemic. World Econ. 2022;45(2):468–522. 10.1111/twec.13183. 34548749 10.1111/twec.13183 PMC8447169

[116] Ugliara Barone MT, Harnik SB, Chaluppe M, et al. Decentralized COVID-19 measures in Brazil were ineffective to protect people with diabetes. Diabetes Metab Syndr. 2020;14(6):1973–8. 10.1016/j.dsx.2020.10.005. 33075740 10.1016/j.dsx.2020.10.005 PMC7538379

[117] Filip R, Gheorghita Puscaselu R, Anchidin-Norocel L, Dimian M, Savage WK. Global Challenges to Public Health Care Systems during the COVID-19 Pandemic: A Review of Pandemic Measures and Problems. J Pers Med. 2022;12(8). 10.3390/jpm12081295. 10.3390/jpm12081295 PMC9409667 36013244

[118] Garelli F, Fushimi E, Rosales N, et al. Non-hybrid glycemic control of type 1 diabetes ambulatory patients. RIAI - Rev Iberoam Autom Inf Ind. 2022;19(3):318–29. 10.4995/RIAI.2022.16652.

[119] Snoswell CL, Taylor ML, Comans TA, Smith AC, Gray LC, Caffery LJ. Determining if Telehealth Can Reduce Health System Costs: Scoping Review. J Med Internet Res. 2020;22(10):e17298. 10.2196/17298. 33074157 10.2196/17298 PMC7605980

[120] Scott Kruse C, Karem P, Shifflett K, Vegi L, Ravi K, Brooks M. Evaluating barriers to adopting telemedicine worldwide: A systematic review. J Telemed Telecare. 2018;24(1):4–12. 10.1177/1357633X16674087. 29320966 10.1177/1357633X16674087 PMC5768250

[121] Macinko J, Woolley NO, Seixas BV, Andrade FB, Lima-Costa MF. Health care seeking due to COVID-19 related symptoms and health care cancellations among older Brazilian adults: the ELSI-COVID-19 initiative. Cad Saude Publica. 2020;36Suppl 3(Suppl 3):e00181920. 10.1590/0102-311x00181920. 33053060 10.1590/0102-311X00181920

[122] Economic Commission for Latin America and the Caribbean (ECLAC). A Digital Path for Sustainable Development in Latin America and the Caribbean.; 2022. https://repositorio.cepal.org/server/api/core/bitstreams/71eb91ed-b241-41c8-9463-d1eaa3b12932/content. Accessed 29 Jan 2024.

